# The Effect of Aquatic Plyometric Training on Jump Performance Including a Four-week Follow-up in Youth Female Volleyball Players

**DOI:** 10.2478/hukin-2022-0058

**Published:** 2022-09-08

**Authors:** Elisa Dell’Antonio, Caroline Ruschel, Marcel Hubert, Ricardo Dantas De Lucas, Alessandro Haupenthal, Helio Roesler

**Affiliations:** 1 Aquatic Biomechanics Research Laboratory, Santa Catarina State University, Florianópolis, Brazil; 2 Physical Effort Laboratory, Federal University of Santa Catarina, Florianópolis, Brazil; 3 Aging, Resources and Rheumatology Laboratory, Federal University of Santa Catarina, Araranguá, Brazil

**Keywords:** power training, aquatic exercise, spike height

## Abstract

This study aimed to analyze the effect of aquatic plyometric training (APT) on jump performance in volleyball players. Twelve female athletes (16.6 ± 0.9 years) were assessed through the following jump tests: spike height (SH), squat jump (SJ), countermovement jump (CMJ) and CMJ with an arm swing (CMJA). Jump height in each test and the eccentric utilization ratio (EUR) were the outcome measures. APT consisted of sets of drop jumps for 6 weeks (2 sessions/week) at a water depth of 0.75 m. Tests were performed at the beginning of a five-week pre-season period, before and after APT, and four weeks later for the follow-up. Repeated measures ANOVAs were used to analyze data and Hedges’ g to estimate effect size (ES). Performance of all jumps did not change from baseline to Pre-APT. Performance improved in SH (p < 0.001, ES: 1.09), the SJ (p = 0.045, ES: 0.76) and the CMJA (p < 0.001, ES: 0.78) after APT when compared to Pre-APT. No changes were observed after the follow-up period. In conclusion, including six weeks of APT in the training routine of youth volleyball players improved performance of a sport-specific task (SH), the SJ and CMJA, with gains preserved after a four-week follow-up.

## Introduction

In volleyball, vertical jumps occur in several game situations, such as the serve, set, attack and block. High level jump performance during these movements may provide decisive advantages when attempting to score. Due to the frequent repetition of those game situations, athletes perform many jumps during a match (Sheppard et al., 2009). Thus, improving jump height is a constant goal in training of these athletes.

Plyometrics is a method used to develop jumping ability in volleyball (Ziv and Lidor, 2010). Previous evidence has shown that plyometrics is effective in improving strength, power, agility, jump height and other performance variables of volleyball players (Silva et al., 2019). Although exercises typically used in plyometrics (e.g. drop jumps) are effective in developing the aforementioned skills, they impose a considerable mechanical load on the players’ musculoskeletal system (Wallace et al., 2010). Thus, surface settings which can reduce this overload, such as sand, grass, synthetic/rubber surfaces, and water, are proposed as alternatives for performing plyometric training.

Aquatic plyometric training (APT) has demonstrated a positive effect on athletic performance (e.g. vertical jump height, velocity, agility) in non-athletes (Held et al., 2019; Robinson et al., 2004) and athletes (Fattahi et al., 2015; Fonseca et al., 2017; Held et al., 2019). The

improvement observed in some variables is equal (Held et al., 2019; Robinson et al., 2004) or even higher (Fattahi et al., 2015; Fonseca et al., 2017) than that obtained in land-based plyometrics. In youth volleyball players, 6- to 8-week APT protocols enhanced agility (Fattahi et al., 2015), muscle strength and vertical jump height (Sargent jump test) (Fattahi et al., 2015; Martel et al., 2005).

The physical properties of water make movements slower and attenuate the reaction forces during jumps (Dell’Antonio et al., 2016). Thus, individuals submitted to jump training in water report less subjective pain perception (Robinson et al., 2004) and less muscle damage is observed (Wertheimer et al., 2018) after APT sessions/programs when compared to land-based training. This may be particularly relevant for training volleyball players who are exposed to repetitive impacts in training routines and competition. These athletes are commonly affected by knee overuse injury (patellar tendinopathy), sometimes presenting with osteoarthritis-related joint alterations (Boeth et al., 2017).

To the best of our knowledge, there is no evidence regarding the effect of APT on performance of volleyball-specific tasks, such as the spike. Improving spike height is particularly important, since it may differentiate between the players´ technical level (Pocek et al., 2020). Attacking directly affects team performance during the match and seems to be the most important skill required by both youth and adult players (Inkinen et al., 2013). It is not known whether improved performance observed after APT is maintained in the post-training period. As such, this study aimed to analyze the effects of six weeks of APT on jump performance, including spike height, of volleyball players and determine whether post-APT improvements remain after a four-week follow-up. We hypothesized that the APT protocol would enhance jump performance in volleyball players, and that, after the follow-up, these gains would be maintained, but at a lower level than that observed immediately post-APT.

## Methods

### Participants

Sample size was calculated based on data from a pilot study analyzing the effect of APT on spike height, using the G*Power software version 3.1.9.2 (University Kiel, Germany). Considering a mean difference of 8 cm between pre- and post-APT, a standard deviation of 10 cm and an alpha of 0.05, twelve participants were required to reach a power of 80%.

Twelve female volleyball players (16.6 ± 0.9 years; 1.72 ± 0.08 m; 63.4 ± 9.7 kg; 7.0 ± 1.9 years of practice; +3.8 ± 0.9 years from peak height velocity (PHV)) participated in the study. All the players took part in national competitions and belonged to the team that won the Brazilian Under-18 Scholar Championship in 2019, with a technical-tactical training routine of 5 weekly sessions of 2.5-hour each. This study was approved by the local ethics committee and all individuals (or their legal guardians when appropriate) provided written informed consent before participating.

### Measures

Participants were instructed to avoid strenuous exercise in the 48 hours preceding a test session and to arrive in a rested and fully hydrated state. All tests were performed at the same time of day to minimize the effects of diurnal biological variation on the results. Participants were verbally and visually instructed on test execution 1 day before the baseline assessment.

Assessments were conducted in the team’s training gymnasium. Participants performed 5 min of light running to warm up and were instructed to execute joint mobilization exercises. After a familiarization period consisting of submaximal jumps, jump tests were carried out in the following order: (1) spike height (SH); (2) countermovement jump (CMJ); (3) squat jump (SJ); and (4) countermovement jump with an arm swing (CMJA). Three valid attempts were collected for all the tests and a minimum of 1 min rest was granted between each repetition.

**Figure 1:**
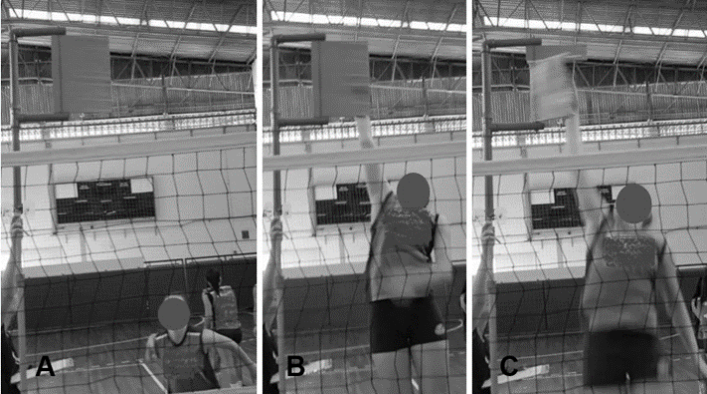
Illustration of the spike height test.A: end of the approach; B: spike jump, aiming to reach the highest possible vane of the device (simulating a spike); C: hand contact and vane displacement.

CMJ, SJ and CMJA height was calculated using the flight time (*height(cm)* = $\left.\left(\frac{9.81\times flight\;time\text{(}s{)}^{2}}{8}\right)\times 100\right),$which was measured with an Elite Jump contact platform (S2 *Sports*, São Paulo, Brazil). The eccentric utilization ratio (EUR) was calculated as the ratio between CMJ and SJ height. A portable device designed and constructed for this study, similar to the Vertec Jump Tester, was used for spike height (SH). The device consists of 80 metal swivel vanes (arranged in 0.5-cm increments) attached to a metal pole. It was positioned near the volleyball net (0.4 m) in the left front position (position 4). Testing facility conditions (e.g., court side, floor surface) were

consistent throughout the tests. Participants performed a full spike movement (3- to 4-step approach, jumping and preparation, a spike and landing) (Tsoukos et al., 2019b), aimed at displacing the highest possible vane (Figure 1). SH was calculated in meters by adding the distance between the height of the base (vane 1) and the highest vane displaced by the participant. The intraclass correlation coefficient (ICC_3,1_; absolute agreement, single measures) for within-trial SH reliability was ≥ 0.96 for all the assessments.

The best execution of each participant in each test and each assessment session was considered for further statistical analysis.

### Design and Procedures

**Figure 2:**
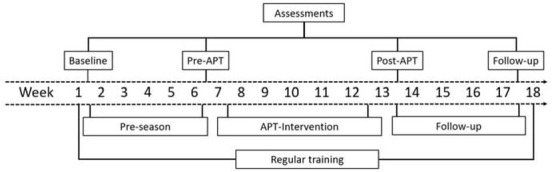
Experimental design. APT: aquatic plyometric training.

Participants were submitted to an initial assessment (Baseline - week 1). After a 5-week period a new assessment (Pre-APT - week 6) was performed. During this period, termed a Pre-season, athletes continued with their regular training routines. Next, a 6-week APT protocol was included in the team’s training routine, and another assessment was carried out after the intervention (Post-APT - week 13). A final assessment was performed four weeks after the end of APT (Follow-up - week 17) (Figure 2).

A total of 12 training sessions were conducted over a 6-week period, with a minimum of 24 hours between sessions. Training was carried out in a swimming pool with water temperature of 27 ± 1ºC and 0.75 m of immersion (equivalent to the immersion between the greater trochanter of the femur and the lateral condyle of the tibia for all the participants). A previous study showed that vertical ground reaction force during the drop jump at this immersion level was significantly lower than on land (Dell’Antonio et al., 2016).

**Figure 3:**
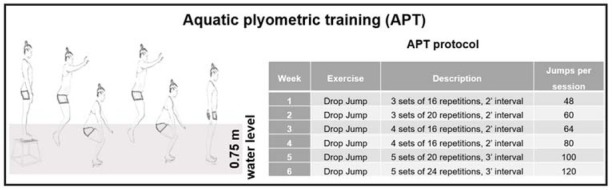
Aquatic plyometric training (APT) protocol.

Each plyometric training session lasted an average of 30 minutes. The training protocol followed the proposal of Fonseca et al. (2017). The volume varied between 48 (first week) and 120 (last week) contacts per session, totaling 944 contacts in 12 APT sessions (Figure 3). Only drop jumps from a box of 0.43 m of height were used in all the training sessions. Players were instructed to contact the ground as fast as possible and jump as high as possible.

During the Pre-season, the team training routine and competition consisted of 23 technical-tactical sessions, and no competitions occurred. During the intervention (APT), 26 technical-tactical training sessions were held, and the team participated in three competitions (36 sets). Between the Post-APT and Follow-up assessments, the team underwent 18 technical-tactical sessions and took part in three competitions (25 sets).

Regular training routine consisted of 5 technical-tactical sessions per week. Training was organized based on the game complexes, with two days for complex 1 (reception, set and attack), two days for complex 2 (serve, block and defense) and one day for a combination of both complexes and their transitions. In addition, resistance training was held three times a week (three sets of 12 to 15 repetitions with 65-70% of 1RM), without power exercises during the study period (from baseline to the follow-up).

### Statistical Analysis

Means and standard deviations were calculated for each variable and data normality was tested using the Shapiro-Wilk’s test. Four one-factor repeated measures analyses of variance (ANOVA) were used to analyze the effect of time on SH, SJ, CMJ, EUR and CMJA. The Bonferroni’s post-hoc test was used for multiple comparisons. Given the objectives and hypotheses of this study, comparisons were made only for the chronological sequence of the assessments, that is, the pre-season (baseline vs. pre-APT), intervention (pre-APT vs. post-APT), and follow-up (post-APT vs. follow-up). Hedges’ g was used for the estimation of the effect size (ES) and values were interpreted as follows (Cohen, 1988): <0.19 = trivial effect; 0.200.49 = small effect; 0.50-0.79 = moderate effect and >0.80 = large effect. The α level was set at 5%, and we used the IBM® SPSS Statistics 20 software (IBM Inc., Armonk, USA) for all statistical procedures.

## Results

Table 1 shows the mean and standard deviations for all the variables at the four assessment points.

**Table 1:** Mean ± standard deviation of the study variables.

Variable	n	Baseline	Pre-APT	Post-APT	Follow-up
SH (m)	12	2.63 ± 0.11	2.63 ± 0.11	2.75 ± 0.11	2.74 ± 0.12
SJ (cm)	12	24.6 ± 3.3	24.9 ± 3.0	27.1 ± 2.8	28.4 ± 3.2
CMJ (cm)	12	26.3 ± 3.9	27.2 ± 3.9	29.0 ± 2.8	29.3 ± 3.4
EUR	12	1.07 ± 0.06	1.09 ± 0.06	1.07 ± 0.04	1.03 ± 0.06
CMJA (cm)	12	31.3 ± 4.0	31.7 ± 3.7	34.7 ± 4.0	34.2 ± 3.8

Abbreviations: APT: aquatic plyometric training; SH: spike height; SJ: squat jump; CMJ: countermovement jump; EUR: eccentric utilization ratio; CMJA: countermovement jump with an arm swing.

The results of ANOVA and post-hoc comparisons are presented in Table 2. Performance of all jumps did not change during the pre-season (from baseline to Pre-APT) (*p* > 0.05). Performance improved in SH (mean difference, MD = 12.0 ± 3.0 cm, *p* < 0.001, ES = 1.09), SJ (MD = 2.2 ± 2.3 cm, *p* = 0.045, ES = 0.76) and CMJA (MD = 3.0 ± 1.3 cm, *p* < 0.001, ES = 0.78) after APT when compared to Pre-APT. No changes compared to Post-APT were observed during the follow-up period (*p* > 0.05).

**Table 2:** Results of ANOVA and Bonferroni’s post-hoc comparisons.

	ANOVA		Post-hoc tests		
	
Outcome	F	*p*	Baseline vs. Pre-APT	Pre-APT vs. Post-APT	Post-APT vs. Follow-up
**SH**	97.128	**<0.001**	1.000	**<0.001** (ES:1.09)	1.000
**SJ**	14.953	**<0.001**	1.000	**0.045** (ES: 0.76)	0.340
**CMJ**	9.130	**<0.001**	0.821	0.156	1.000
**EUR**	2.526	0.074	1.000	1.000	0.311
**CMJA**	16.453	**<0.001**	1.000	**<0.001** (ES: 0.78)	1.000

Abbreviations: APT: aquatic plyometric training; SH: spike height; SJ: squat jump; CMJ: countermovement jump; EUR: eccentric utilization ratio; CMJA: countermovement jump with an arm swing; ES: effect size (Hedges’ g).

## Discussion

The aim of this study was to analyze the effect of a 6-week APT program on jump performance of youth volleyball players. Our main findings demonstrate that adding APT to the training routine of athletes improved SH, SJ and CMJA performance. Four weeks after the end of APT, the performance gains were maintained.

### Pre-season

After the pre-season which included the first five weeks of the annual training program, jump performance did not change. During this time, training sessions were aimed at improving technical skills and overall physical conditioning (endurance and muscle strength, aerobic exercises, and coordination). Although this approach has proven to be important in improving game performance with regard to serving, reception, setting and attacking (Gabbett, 2008), it seems to be unable to change vertical jump height in six (Trajković et al., 2012) or twelve weeks (Gabbett, 2008).

### Experimental

After APT, SH improved substantially (average difference of 12 cm). Spike height is very important for athletic performance and professional practice (Stanganelli et al., 2008). A higher attack reach results in better offensive actions at the net, is a determinant in scoring points and winning games, and is also associated with higher technical levels (Gabbett and Georgieff, 2007). Moreover, SH is considered for players’ selection processes (Tsoukos et al., 2019a) which favor athletes with greater attack reach. The improvement observed for SH was far higher than that found for the other jumps (2-4 cm). This reinforces the need to analyze sport-specific task performance to better understand the effect of interventions in this population.

The enhanced performance in non-specific vertical jumps (i.e. SJ and CMJA) observed after APT corroborates previous findings reporting the positive effect of both land- (Asadi and Ramírez-Campillo, 2016; Pereira et al., 2015) and water-based (Fattahi et al., 2015; Martel et al., 2005) plyometrics on jump height. The effect size of land-based plyometric training on jump height in female athletes varies according to the type of the jump, duration of the training program and the level of participants (Stojanović et al., 2017). In our study, we found a moderate effect size for the SJ and CMJA, even with a short APT period. The magnitude of changes in girls seems to be influenced by maturational status, where the effects seem to be less significant after the maturity offset (i.e. PHV) (Davies et al., 2019). This underscores the results achieved by the proposed intervention, given that participants were, on average, 4 ± 1 years post PHV.

An increase in jump performance is related to enhanced muscle power (Makaruk and Sacewicz, 2010). In the SJ, where only concentric muscle actions occur during the propulsion phase, the improved performance may be related to an increase in muscle strength. Earlier studies analyzing the effects of APT in volleyball players reported an increase in the concentric peak torque of knee extensors (Martel et al., 2005) and maximum leg press strength (Fattahi et al., 2015). Performance gains in the CMJA and SH, which involve eccentric and concentric muscle actions, may be due to the neural adaptations caused by training (Asadi and Ramírez-Campillo, 2016). The adaptations arising from plyometric training would increase the capacity of muscles to produce strength and power (Guilhem et al., 2010). This would be possible due to an increased neural drive of the agonist muscles, intramuscular coordination, and the ability of using the stretch-shortening cycle, as well as changes in the muscle-tendon stiffness and muscle architecture (Markovic and Mikulic, 2010). Although the eccentric load (i.e., braking force) in water is reduced as compared to land (Dell’Antonio et al., 2016), one could expect the occurrence of structural changes in the muscle-tendon system and adaptation in the central nervous system (Guilhem et al., 2010) as an effect of APT. Future studies are warranted to elucidate whether such neuromuscular adaptation occurs and how it relates to the performance improvements observed herein.

In drop jumps, such as those used in the intervention, longer contact times are associated to greater force and higher jump height (Walsh et al., 2004). In water, the contact time is longer than on land, even at low immersion levels (Dell’Antonio et al., 2016). This may have contributed to gains in jump performance after APT. It should be noted that participants were engaged in resistance training three times a week during the study, which likely had a positive influence on the results (Marques et al., 2008). Additionally, since the team’s training routine was maintained during the intervention, performance gains cannot be attributed exclusively to APT. However, the regular technical-tactical training routine by itself (Pereira et al., 2015) or carried out concurrently to resistance training (Marques et al., 2008) seems to be insufficient to enhance CMJ performance in volleyball players.

The intervention period did not change the EUR, corroborating the results of a previous study which analyzed the effect of land-based plyometrics in a group of college-aged males (Hawkins et al., 2009). The duration of the intervention and/or the aquatic environment may not have been able to produce significant improvements in the ratio between CMJ and SJ height.

### Follow-up

The gains in SH, SJ and CMJA observed after APT were maintained four weeks after the end of the intervention. To the best of our knowledge, no other study investigating the effects of APT on athletic performance included a follow-up period. Our results demonstrate the potential of this type of intervention for volleyball players, given that performance was maintained for around one month after the last APT session, within the competitive period.

In athletes, most studies involving plyometrics address only the immediate effects after intervention, and information on the maintenance (or not) of changes after a certain period is scarce (Ramirez-Campillo et al., 2018). This information is particularly important in the sports training field to assist coaches to choose when to apply the intervention throughout the team’s preparation season. In land-based training, the positive effects of a 10-week plyometric program on jump performance were maintained for 16 weeks in youth basketball players, only by continuing the technical-tactical training regimen (Santos and Janeira, 2011). Stanganelli et al. (2008) reported that jump performance gains (SJ, CMJ, attack jump and reach and blocking) achieved after a 9-week program (not including plyometrics) of physical, technical-tactical and resistance training and games were maintained in the nine ensuing weeks. Based on these results and those obtained in our study, technical-tactical and resistance training, along with games (competitions) seem to be sufficient to maintain physical performance for a period of time.

Despite the novelty of the data presented here, some limitations should be noted. All the APT sessions were held after technical-tactical training, despite its being recommended before (Ramirez-Campillo et al., 2020). This was done to influence the team’s training routine as little as possible, since the transition from the swimming pool to the court would take considerable time and could affect total session time. The authors recognize that generalizing the main outcomes may be limited by the small number of participants and the absence of a control group. Even with the inclusion of the pre-APT period as an alternative to decrease bias caused by the single-group design when interpreting the effects of APT, we cannot rule out a possible order effect (control-intervention) on the results. However, the small number of players in a volleyball team hinders the creation of different subgroups with a sampling size that guarantees sufficient statistical power. Although analyzing players from different teams may minimize this problem, it would mean adding important confounding variables, since the training routine would not be the same. It is also important to underscore that the current findings should be extrapolated to male or older female volleyball players with caution, since only young women were analyzed here.

In conclusion, a 6-week aquatic plyometric training protocol with 0.75 m immersion improved squat jump, countermovement jump with an arm swing and spike height performance in female volleyball players. Four weeks after the intervention, these gains were maintained. Our results suggest that APT can be considered an option for physical training of volleyball players in a setting where the mechanical load on landing is reduced. This would enable coaches to increase the load of other types of stimuli, such as those included in technical-tactical training. The proposed protocol, consisting of 30-min sessions twice a week for a 6-week period, which is feasible to be implemented at different times during the season, promoted important improvements in the athletes’ jump performance.
